# Supplementary material for: “You know that we travel a lot”: Mobility narratives among female sex workers living with HIV in Tanzania and the Dominican Republic
Source: PLOS Glob Public Health. 2024 Jul 5;4(7):e0003355. doi: 10.1371/journal.pgph.0003355 (PMC11226099; doi:10.1371/journal.pgph.0003355)
Supplement: S1 Table — (DOCX) [file pgph.0003355.s002.docx]

***S1 Table. Supplemental mobility narratives of FSW in Tanzania and the Dominican Republic.***

| ***Mobility narratives in Tanzania*** | ***Mobility narratives in the Dominican Republic*** |
| --- | --- |
| ***Mary, whose clients influence travel***  Mary, a Tanzanian woman, traveled both to visit family and for sex work. Her mobility was an economic decision. Her trips were often led by or motivated by clients, and often unplanned. She also connected with family when traveling. She felt supported when she traveled because she had family connections if problems arose with a client. Although she often did not prepare much, she told fellow workers about her trips and noted clients’ license plate numbers before traveling. She also made sure she brought her clinic card with her. She traveled for sex work to places where people did not know her or know that she is living with HIV. She said that if one stays in one place too long, clients “no longer pay well,” so she moved to “find someone who can pay well.” Her trips for sex work were not long – perhaps one, two, or four days – and not frequent. “For instance, I go to Ruaha Mbuyuni (100 km east of Iringa city) and stay there for two days and do business and then come back,” she said. | ***Camila, who work on the street (“de la calle”) in local towns***  Camila, from the DR, traveled to local towns outside Santo Domingo (e.g., Bonao, a northern town), where she would meet primarily Dominican clients on the streets, in parking lots, parks, and hotels. She stated, “men driving through these towns, including local men who lived in one of these towns, know that they could find a woman there and buy sex.” She would then go to nearby hotels with these clients. She received money and other types of resources from them, especially those she met in the park, who were older, retired men. She said, “I have known some of these men for a long time. They sometimes give me 100 pesos (~$1.85 USD) and pay for my meals.” She called them “amigos” during the interview, describing how she could receive a small amount of money from them for a sexual favor to purchase food. She explained the desperateness she would feel in certain moments when her “amigos” did not show up. She also traveled to hotels in these towns in hopes of meeting other male clients. |
| ***Martha, who moves locally to visit family and seek healthcare***  While Martha had not recently traveled outside of Iringa, she had moved away from Mafinga (destination along the highway just south of Iringa city) between the first and second interview. She did not want to return to Mafinga, but she remained in contact with fellow workers who visited her after she moved. She had recently traveled within Iringa to visit family members such as her mother, grandmother, or sisters. She went for funerals, when her grandmother was sick, or at Christmas as part of a family tradition. Martha described how her visits to see her sisters and family often intersected with economic needs. Her sisters would send her bus fare so that she could visit them. They would give her resources - flour, rice, oil, money. “They help me with what they can afford,” she said. As she now lived far from the clinic where she sought care, she would start her journey to the clinic the day before her appointment. Some days, she lacked sufficient fare for the bus. When she had fare, she would take public transport to her sister’s house and then go to the clinic by foot the following day. She would not return immediately, but often stay at her sister’s house after taking her medication. In fact, she had started to leave her clinic card at her sister’s house. Others in Martha’s life (her husband, friends, sisters, and other FSW) were also mobile. Such trips were often business related. Her sister, for example, had a business in Dar-es-Salaam. During her sisters’ trips, she would cooperate with them by staying with the children and taking care of them. | ***Sophia, who meets local clients via online apps***  “Codigo” is what the chicas call this type of sex work where they find someone via an online app (like Badoo) and then travel to meet the male clients somewhere like a nightclub or pool hall. “I sometimes use an app…that tells me how distant the person is from me and I can connect with them using the app…If it is close, I go to meet them usually somewhere in the city,” she said. She meets new clients this way. She also has regular clients who she contacts via phone to set up a place and time to meet. She said, “I have a day job but do not make enough money so I engage in sex work too.” She does not always stay overnight with clients; she takes a cab back home. Main reason is economic. She says she does not make enough money to make ends meet. |
| ***Shani, the experienced, seasonal traveler in search of anonymity***  Shani considered herself “experienced,” which meant that she knew when and where to go in search of business, or sex work. “We know; we have been doing this job for quite a long time, so we are experienced. So, once I am here I know at Morogoro [northeast of Iringa] is special season for this kind of business [sesame and rice harvesting] or if I go to a certain mining center, gold is available so I will make money,” she said. If she traveled to these specific locations, Dodoma during grape season or a parliamentary session, Mtera when fish are found in excess – she knew she would leave with money. “We are after fishermen’s money,” she said. She would not stay in these specific locations for long periods – perhaps a few days or a week – to remain “new to the place” and not become “familiar.” She did not engage in sex work at home. “I can’t do what I do when I am away in Iringa, it is my home.” She would decide where to go based on feedback from fellow workers. “We usually inform one another,” she said. She might string a few locations together during a trip. For example, Mtera to Dodoma to Mbeya to Morogoro before returning to Iringa. During her travel she would work as a bar worker, but she preferred not to be paid for that work. It gave her autonomy and flexibility to leave when she wanted, and “the salary isn’t much anyway – I can make that [a month’s salary] in one day.” Her family members also traveled often – from her aunt to her husband to her uncle, who traveled to visit her, take care of her, or take her to the health clinic when necessary. | ***Yari, who travels to distant towns***  Yari talked about going to distant towns (e.g,, Higüey, east of Santo Domino) for anonymity. She could avoid all the gossip by going to towns where no one knew her. She did not have family in these places either. She would stay for 2 months at a time, come home for 15 days and then go back. She lived with other FSW in a hotel. She traveled with a group of women sometimes. They provided her a room for sex work. She also goes to the hotels in these towns to pick up men there. She explained that she travels for economic reasons, saying that sex work was quick money and her family counted on it as well. She was financially providing for her family. They all knew she was engaged in sex work. |
| ***Zia, who moves locally***  Zia worked “on the road” in Iringa, where she often changed work environments and moved locally to find and follow clients. In this setting, clients were often people who “are passing by,” making it difficult to negotiate with them. She had recently traveled within and outside of Iringa to see family (e.g., elsewhere in Iringa region to see her mother, Dar-es-Salaam to visit family, or Moshi in the northern part of the country for her sister’s marriage) and “to have a good time” in Mafinga. Her favorite place to visit was her mother’s home, but she did not exchange sex while traveling there. “I cannot go there and do that, that is my community, I don’t like my mother to know what I do,” she said. Trips such as her recent trip to Moshi for her sister’s marriage required bus fare, but she did not face any problems during travel. She traveled with her father to Moshi, and since the journey took only one day, the preparations required were minimal. | ***Elianny, who goes to the rural side (“el campo”) to meet clients***  Elianny calls her mobile sex work ‘El chiripeo,’ a local Dominican term for sex work. She leaves by bus in the morning to the rural areas (e.g. El Castillo). She leaves on Friday and returns on Monday. The main motivation for her mobility is economic. She says the economic situation is worse for her clients in the campo now (“there is no money”), and so now she must sometimes stay 2-3 weeks to get more money. The campo where she goes is primarily based on the cacao economy and the harvest was bad for years, leading to decreased prices and greater poverty in the area. As a result, she also says that now there are more people “con vicios” and increasingly use drugs. Elianny also describes the desire for anonymity, as her children and family do not know she sells sex. When she travels, she stays at a room at a hotel and the hotel owner gets a room fee for every client she brings into the room. She works independently, travels alone, and controls her schedule, she says. She also has a few regular clients who she arranges to meet prior to her travels. |
