# Supplementary material for: “You know that we travel a lot”: Mobility narratives among female sex workers living with HIV in Tanzania and the Dominican Republic
Source: PLOS Glob Public Health. 2024 Jul 5;4(7):e0003355. doi: 10.1371/journal.pgph.0003355 (PMC11226099; doi:10.1371/journal.pgph.0003355)
Supplement: Attachment — Submitted filename: Reviewer Feedback.docx [file pgph.0003355.s003.docx]

**Manuscript comments[ Manuscript Number PGPH-D-23-01890]**

1. The claims made by this study are relevant for future research however, they will be better placed if they highlight their link with access to health care. Nonetheless, these are significant as they can contribute to the development of contextual interventions for FSW who are highly mobile and those who are localized.
2. The claims are properly placed in the context of previous literature which in essence informs the conceptualization of the study.
3. Analysis supports the data however, some themes are emphasized on and given the attention necessary as compared to other (e.g. anonymity and familiarity vis emergent typologies).
4. There is no need for protocol attachment for this article.
5. The authors are encouraged to resubmit.
6. The manuscript will not be clearly understood by non-specialist however below are suggestion made to improve the quality and ease of read by all who might be interested despite their level of expertise in the field.

Line 9 under introduction: For grammatical purposes, I recommend that you only state systematic review and not systematic review and study as stated because the systematic review is the study and not two different activities.

Line 8 under conceptualization: Because special and temporal are two different concepts, it would have been best to differentiate the examples rather than lamp them together.

Study objectives are clearly stated.

Is there a reason for the differentiation of participants according to their viral load/ suppression? Please state the justification.

The sample description is a little confusing and needs to be simplified. Although the authors stated the 40 participants in total, it is not clear how many participated per round except that 27 participated were included in the first phase of analysis. In the same paragraph, the authors state that 40 participants were qualitatively interviewed but when the inclusion for analysis was made, those who described mobility in the quantitative study were included. The statement said “either or” which implies that data from the quantitative study which is not part of this report was sought. Please clarify this properly and also clarify if it was the same 27 participated in round 2 (qualitative rounds) adding an explanation of the 13 seemingly excluded group.

Under the narrative section particularly in describing the characteristics, the authors should compare similar aspects, so the differences are clear. Example: “In Tanzania, mobility included travel to visit family (reason for mobility)” whereas in the DR segment, the discussion is on the nature of travel and the reason for mobility is not stated to make a comparison.

Under anonymity and familiarity, I suggest that particularly for familiarity, the authors should highlight that FSW perceive this in both a positive and negative light. This will make the narrative clear to the reader rather than being independently stated and no link made.

The authors sought to “inform future contextualized research on FSW’s mobility and HIV care and treatment interventions”. It is noted that proposals were made on how the themes from the narratives can be addressed however, it would be more appropriate if these we to be done for all the themes that emerged.

Compiled by D R Phetlhu
