# Supplementary material for: “You know that we travel a lot”: Mobility narratives among female sex workers living with HIV in Tanzania and the Dominican Republic
Source: PLOS Glob Public Health. 2024 Jul 5;4(7):e0003355. doi: 10.1371/journal.pgph.0003355 (PMC11226099; doi:10.1371/journal.pgph.0003355)
Supplement: Attachment — Submitted filename: Review_recommendations.docx [file pgph.0003355.s004.docx]

**Review “You know that we travel a lot”: Mobility narratives among female sex workers living with HIV in Tanzania and the Dominican Republic”**

This qualitative study describes the mobility narratives among female sex workers in two settings, Tanzania and Dominican Republic.

The study is highly informative in gaining further understanding into the motivations and patterns of mobility of FSWs, which further informs FSW typology. This is crucial, as previous quantitative studies were not sufficiently able to provide accurate typology of FSW mobility patterns.

The paper is well-written and the analysis is thorough. I recommend publication.
