# Supplementary material for: “You know that we travel a lot”: Mobility narratives among female sex workers living with HIV in Tanzania and the Dominican Republic
Source: PLOS Glob Public Health. 2024 Jul 5;4(7):e0003355. doi: 10.1371/journal.pgph.0003355 (PMC11226099; doi:10.1371/journal.pgph.0003355)
Supplement: Attachment — Submitted filename: PlosGH_manuscript_anonymized_reviewer responses_07FEB2024.docx [file pgph.0003355.s005.docx]

Dear Editorial Board,

The authors thank the reviewers for their thoughtful review of the manuscript and their excellent suggestions for places to strengthen the manuscript. We have responded to each of their comments in depth below, identifying specific places where changes were made and content added to address the comment and improve or clarify the manuscript as necessary. We have reviewed the reference list as requested and addressed formatting issues where necessary. We have also addressed the additional requirements requested.

Please note that in the responses below, the page numbers and line numbers referenced refer to those page numbers/line numbers in the tracked version uploaded. The line numbers may be slightly different in the clean version, although the documents are identical.

We believe that with these revisions and requested additions from the reviewers, the revised manuscript has been strengthened and has the opportunity to contribute to a greater understanding of mobility typologies among female sex workers living with HIV.

Thank you for your consideration of this revised manuscript. We look forward to hearing from you.

Sincerely,

The co-author team

**Comments from editor:**

1. Please review your reference list to ensure that it is complete and correct. If you have cited papers that have been retracted, please include the rationale for doing so in the manuscript text, or remove these references and replace them with relevant current references. Any changes to the reference list should be mentioned in the rebuttal letter that accompanies your revised manuscript. If you need to cite a retracted article, indicate the article’s retracted status in the References list and also include a citation and full reference for the retraction notice.

Response: We have followed these recommendations and reviewed the reference list accordingly. Please note that we have added four references to the source of the basemap files for the figures created for this manuscript.

2. Please include a complete copy of PLOS’ questionnaire on inclusivity in global research in your revised manuscript. Our policy for research in this area aims to improve transparency in the reporting of research performed outside of researchers’ own country or community. The policy applies to researchers who have travelled to a different country to conduct research, research with Indigenous populations or their lands, and research on cultural artefacts. The questionnaire can also be requested at the journal’s discretion for any other submissions, even if these conditions are not met.  Please find more information on the policy and a link to download a blank copy of the questionnaire here: <https://journals.plos.org/globalpublichealth/s/best-practices-in-research-reporting>. Please upload a completed version of your questionnaire as Supporting Information when you resubmit your manuscript.

Response: We have completed this questionnaire and uploaded it as supporting information as part of our submission.

3. Please provide separate figure files in .tif or .eps format only and remove any figures embedded in your manuscript file. Please also ensure all files are under our size limit of 10MB.

Response: We have uploaded the figure files in .tif format and all are under 10MB. There are no embedded figures in the manuscript file.

For more information about figure files please see our guidelines:

<https://journals.plos.org/globalpublichealth/s/figures> 
<https://journals.plos.org/globalpublichealth/s/figures#loc-file-requirement>

4. We have noticed that you have a list of Supporting Information legends in your manuscript. However, there are no corresponding files uploaded to the submission. Please upload them as separate files with the item type 'Supporting Information'.

Response: We reference a supplementary material in the results section that is currently uploaded as Supporting Information: PlosGH_supplementaltable_20SEPT2023.docx. We also had uploaded the figures separately and have reviewed to ensure that all documents have been uploaded accordingly as part of this resubmission.

5. Some material included in your submission may be copyrighted. According to PLOS’s copyright policy, authors who use figures or other material (e.g., graphics, clipart, maps) from another author or copyright holder must demonstrate or obtain permission to publish this material under the Creative Commons Attribution 4.0 International (CC BY 4.0) License used by PLOS journals. Please closely review the details of PLOS’s copyright requirements here: PLOS Licenses and Copyright. If you need to request permissions from a copyright holder, you may use PLOS's Copyright Content Permission form.

Please respond directly to this email or email the journal office and provide any known details concerning your material's license terms and permissions required for reuse, even if you have not yet obtained copyright permissions or are unsure of your material's copyright compatibility.

Response: Thank you for this point. We have made the necessary updates to the figure legends to clarify that our figures use CC BY 4.0 permission.

Potential Copyright Issues:

Figs 2 & 3: please (a) provide a direct link to the base layer of the map (i.e., the country or region border shape) and ensure this is also included in the figure legend; and (b) provide a link to the terms of use / license information for the base layer image or shapefile. We cannot publish proprietary or copyrighted maps (e.g. Google Maps, Mapquest) and the terms of use for your map base layer must be compatible with our CC-BY 4.0 license.

Note: if you created the map in a software program like R or ArcGIS, please locate and indicate the source of the basemap shapefile onto which data has been plotted.

Response: We created the maps using ArcGIS. We have updated the legend to include information about the basemap as described above. Updated figure legends are below:

Maps were created using ArcGIS using basemap shapefiles from the UN Humanitarian Data Exchange (https://data.humdata.org/), which is covered under the Creative Commons Attribution 4.0 International (CC BY 4.0).

The updated Figure legends are as follows (with associated references):

Figure 1. Districts, cities, and/or towns in Tanzania named in participants’ descriptions of mobility. Maps were created using ArcGIS using basemap shapefiles from the UN Humanitarian Data Exchange, which is covered under the Creative Commons Attribution 4.0 International (CC BY 4.0). Specific basemap shape files were the United Republic of Tanzania - Subnational Administrative Boundaries (48) and the HOTOSM Tanzania Roads (OpenStreetMap Export) (49).

Figure 2. Municipalities, towns, and/or neighborhoods in the Dominican Republic named in participants’ descriptions of mobility. Municipalities, towns, and/or neighborhoods in the Dominican Republic named in participants’ descriptions of mobility. Maps were created using ArcGIS using basemap shapefiles from the UN Humanitarian Data Exchange, which is covered under the Creative Commons Attribution 4.0 International (CC BY 4.0). Specific basemap shape files were the Subnational Administrative Boundaries by Oficina national de Estadistica, OCHA Field Information Services Section (50) and the Dominican Republic Roads (OpenStreetMap Export) by Humanitarian OpenStreetMap Team (HOT) (51).

References:

48. Humanitarian Data Exchange. United Republic of Tanzania - Subnational Administrative Boundaries [Internet]. 2018 [cited 2024 Feb 12]. Available from: https://data.humdata.org/dataset/cod-ab-tza

49. Humanitarian Data Exchange. HOTOSM Tanzania Roads (OpenStreetMap Export) [Internet]. 2020 [cited 2024 Feb 12]. Available from: https://data.humdata.org/dataset/hotosm_tza_roads

50. Dominican Republic - Subnational Administrative Boundaries - Humanitarian Data Exchange [Internet]. [cited 2024 Feb 12]. Available from: https://data.humdata.org/dataset/cod-ab-dom?

51. Humanitarian Data Exchange. Dominican Republic Roads (OpenStreetMap Export) - Humanitarian Data Exchange [Internet]. 2020 [cited 2024 Feb 12]. Available from: https://data.humdata.org/dataset/hotosm_dom_roads

If your map was obtained from a copyrighted source please amend the figure so that the base map used is from an openly available source. Alternatively, please provide explicit written permission from the copyright holder granting you the right to publish the material under our CC-BY 4.0 license.

Response: N/A. See response above.

Please note that the following CC BY licenses are compatible with PLOS license: CC BY 4.0, CC BY 2.0 and CC BY 3.0, meanwhile such licenses as CC BY-ND 3.0 and others are not compatible due to additional restrictions.

Response: Thank you for this clarification. The CC BY licenses are CC BY 4.0.

If you are unsure whether you can use a map or not, please do reach out and we will be able to help you. The following websites are good examples of where you can source open access or public domain maps: 
* U.S. Geological Survey (USGS) - All maps are in the public domain. (<http://www.usgs.gov>) 
* PlaniGlobe - All maps are published under a Creative Commons license so please cite “PlaniGlobe, <http://www.planiglobe.com>, CC BY 2.0” in the image credit after the caption. (<http://www.planiglobe.com/?lang=enl>) 
* Natural Earth - All maps are public domain. (<http://www.naturalearthdata.com/about/terms-of-use/>)
"

Reviewers' comments:

5. Review Comments to the Author

Please use the space provided to explain your answers to the questions above. You may also include additional comments for the author, including concerns about dual publication, research ethics, or publication ethics. (Please upload your review as an attachment if it exceeds 20,000 characters)

Reviewer #1: The work has the potential to contribute to the field of HIV and FSW thus relevant. Detailed comments and recommendations are given in the attached document to increase the quality of the paper to the standard that is acceptable measured by ease of understanding by the reader.

Response: Thank you very much for your review of this manuscript. We have responded to your detailed comments and recommendations below.

Reviewer #2: Review “You know that we travel a lot”: Mobility narratives among female sex workers living with HIV in Tanzania and the Dominican Republic”

This qualitative study describes the mobility narratives among female sex workers in two settings, Tanzania and Dominican Republic.

The study is highly informative in gaining further understanding into the motivations and patterns of mobility of FSWs, which further informs FSW typology. This is crucial, as previous quantitative studies were not sufficiently able to provide accurate typology of FSW mobility patterns.

The paper is well-written and the analysis is thorough. I recommend publication.

Response: Thank you very much for your review of this manuscript. We appreciate the positive feedback and recommendation for publication!

**Reviewer #1:**

**Manuscript comments[ Manuscript Number PGPH-D-23-01890]**

1. The claims made by this study are relevant for future research however, they will be better placed if they highlight their link with access to health care. Nonetheless, these are significant as they can contribute to the development of contextual interventions for FSW who are highly mobile and those who are localized.

Response: Thank you for this important point. We agree that these findings are particularly relevant given their potential effects on people’s access to healthcare. We raise this point in the introduction on page 4 (lines 2-6) and have more clearly integrated this point into the abstract (see page 2, line 23-page 3, line 1) and the discussion section on page 22 (lines 19-23).

2. The claims are properly placed in the context of previous literature which in essence informs the conceptualization of the study.

Response: Thank you very much for this comment.

3. Analysis supports the data however, some themes are emphasized on and given the attention necessary as compared to other (e.g. anonymity and familiarity vis emergent typologies).

Response: Thank you very much for this important point. We have reviewed the results and discussion sections to ensure that we provide sufficient detail to each of the important themes. The emergent typologies reflect a combination or pattern of spatial, temporal, and social factors that characterized FSW’s mobility experiences. For the sake of space, we did not elaborate on these patterns but instead use the visual representation to depict this. This visual representation summarizes the patterns that emerged across FSW’s narratives (summarized in Section 3.1.1, 3.1.2, or Supplemental Table 1). We clarify this in the results section (see page 19, lines 1-4) and spotlight these findings in the discussion section as a first step that needs further interrogation in future research and programming (See page 22, lines 11-page 23, line 6).

4. There is no need for protocol attachment for this article.
5. The authors are encouraged to resubmit.
6. The manuscript will not be clearly understood by non-specialist however below are suggestion made to improve the quality and ease of read by all who might be interested despite their level of expertise in the field.

Response: Thank you very much for these detailed comments. We have responded to each of them below in turn and believe that with these revisions, the manuscript is now clearer for readers.

Line 9 under introduction: For grammatical purposes, I recommend that you only state systematic review and not systematic review and study as stated because the systematic review is the study and not two different activities.

Response: Thank you for raising this grammatical point. In this sentence, we are referring to results from two different studies – one was a systematic review, while the other was a study conducted in Zimbabwe. We have reworded this sentence for clarity (Introduction, page 4, lines 9-12). It now reads: **“However, a study in Zimbabwe, consistent with findings from a recent systematic review, demonstrated how associations vary with context, citing mixed evidence on relationships between mobility and antiretroviral therapy (ART) adherence and viral load and calling for improved measurement of FSW’s mobility (15,19).”**

Line 8 under conceptualization: Because special and temporal are two different concepts, it would have been best to differentiate the examples rather than lamp them together.

Response: Thank you for this recommendation. We have disentangled the two as recommended. See Section 1.1, page 5, lines 8-10. It now reads: **“Spatial characteristics of mobility include, for example, destination(s) of travel, while temporal characteristics can include duration or frequency of travel.”**

Study objectives are clearly stated.

Response: Thank you very much!

Is there a reason for the differentiation of participants according to their viral load/ suppression? Please state the justification.

Response: This qualitative study was conducted as part of a larger parent study focused on understanding HIV-related stigma and social cohesion among female sex workers living with HIV. As part of this larger parent study’s aims, purposive recruitment based on viral load was conducted to explore the interplay between HIV and sex work-related stigma and social cohesion in relation to HIV services and outcomes. We have incorporated this into the manuscript on page 7, lines 14-16.

The sample description is a little confusing and needs to be simplified. Although the authors stated the 40 participants in total, it is not clear how many participated per round except that 27 participated were included in the first phase of analysis. In the same paragraph, the authors state that 40 participants were qualitatively interviewed but when the inclusion for analysis was made, those who described mobility in the quantitative study were included. The statement said “either or” which implies that data from the quantitative study which is not part of this report was sought. Please clarify this properly and also clarify if it was the same 27 participated in round 2 (qualitative rounds) adding an explanation of the 13 seemingly excluded group.

Response: Thank you for identifying this area of confusion. We have clarified the methods section to more clearly explain the sample. Individuals who participated in the qualitative interviews were recruited from the larger parent cohort study. As part of this parent cohort study, there were three rounds of quantitative surveys conducted. There were two rounds of qualitative interviews, and we reviewed transcripts from round 1 and 2 as well as responses to mobility-related questions in the quantitative surveys to determine whether interviews were deemed eligible for analysis. We conducted our analysis in two phases as outlined in the manuscript. We have incorporated these clarifying pieces into the manuscript on page 6, lines 17-18; page 7, line 22-page 8, line 7.

Under the narrative section particularly in describing the characteristics, the authors should compare similar aspects, so the differences are clear. Example: “In Tanzania, mobility included travel to visit family (reason for mobility)” whereas in the DR segment, the discussion is on the nature of travel and the reason for mobility is not stated to make a comparison.

Response: Thank you for this point. We have revised this section to more clearly draw comparisons between the two contexts, where relevant. For example, please see page 11, line 24 or page 12, lines 19-21 or page 14, lines 12-16. We have in other places clarified where themes were true in both settings. For example, please see page 15, lines 1-2.

Under anonymity and familiarity, I suggest that particularly for familiarity, the authors should highlight that FSW perceive this in both a positive and negative light. This will make the narrative clear to the reader rather than being independently stated and no link made.

Response: Thank you for this recommendation. We have incorporated this into the paragraph on page 17, lines 4-6, to elucidate the linkage between anonymity and familiarity and how FSW perceived familiarity in both positive and negative ways. Please also see page 17, lines 24-25.

The authors sought to “inform future contextualized research on FSW’s mobility and HIV care and treatment interventions”. It is noted that proposals were made on how the themes from the narratives can be addressed however, it would be more appropriate if these we to be done for all the themes that emerged.

Response: Thank you for this point. The discussion section draws on key themes that emerged from the qualitative interviews overall (both phases of analysis), not only the narrative analysis and the narratives spotlighted in the manuscript itself. In the discussion section, we have incorporated recommendations related to HIV care and treatment that address multiple themes emerging from the qualitative interviews, including financial insecurity and economic motivations for mobility; social relationships and ways to foster social cohesion; the interplay between anonymity and familiarity; among others. We have clarified the language in the discussion section to make it clear that the themes discussed pertain to the overall findings of this study and were not limited to the narratives spotlighted in the manuscript. Please see, for example, page 19, line 10; page 20, line 1; page 22, line 4; page 22, line 11; page 23, line 13.

Compiled by D R Phetlhu

**Reviewer #2**

**Review “You know that we travel a lot”: Mobility narratives among female sex workers living with HIV in Tanzania and the Dominican Republic”**

This qualitative study describes the mobility narratives among female sex workers in two settings, Tanzania and Dominican Republic.

The study is highly informative in gaining further understanding into the motivations and patterns of mobility of FSWs, which further informs FSW typology. This is crucial, as previous quantitative studies were not sufficiently able to provide accurate typology of FSW mobility patterns.

The paper is well-written and the analysis is thorough. I recommend publication.

Response: Thank you very much for your review of this manuscript. We appreciate the positive feedback and recommendation for publication!
